# Characterization of the First Virulent Phage Infecting *Oenococcus oeni*, the Queen of the Cellars

**DOI:** 10.3389/fmicb.2020.596541

**Published:** 2021-01-13

**Authors:** Cécile Philippe, Amel Chaïb, Fety Jaomanjaka, Olivier Claisse, Patrick M. Lucas, Johan Samot, Christian Cambillau, Claire Le Marrec

**Affiliations:** ^1^University of Bordeaux, ISVV, EA4577 Œnologie, Villenave d’Ornon, France; ^2^INRA, ISVV, USC 1366 Oenologie, Villenave d’Ornon, France; ^3^Architecture et Fonction des Macromolécules Biologiques, Aix-Marseille Université, Campus de Luminy, Marseille, France; ^4^Architecture et Fonction des Macromolécules Biologiques, Centre National de la Recherche Scientifique (CNRS), Marseille, France; ^5^Bordeaux INP, ISVV, EA4577 Œnologie, Villenave d’Ornon, France

**Keywords:** lactic acid bacteria, phage (bacteriophage), phage phylogenetics, lytic phage, *Oenococcus*, fermentation, wine, grapes

## Abstract

There has been little exploration of how phages contribute to the diversity of the bacterial community associated with winemaking and may impact fermentations and product quality. Prophages of *Oenococcus oeni*, the most common species of lactic acid bacteria (LAB) associated with malolactic fermentation of wine, have been described, but no data is available regarding phages of *O. oeni* with true virulent lifestyles. The current study reports on the incidence and characterization of the first group of virulent oenophages named Vinitor, isolated from the enological environment. Vinitor phages are morphologically very similar to siphoviruses infecting other LAB. Although widespread during winemaking, they are more abundant in musts than temperate oenophages. We obtained the complete genomic sequences of phages Vinitor162 and Vinitor27, isolated from white and red wines, respectively. The assembled genomes shared 97.6% nucleotide identity and belong to the same species. Coupled with phylogenetic analysis, our study revealed that the genomes of Vinitor phages are architecturally mosaics and represent unique combinations of modules amongst LAB infecting-phages. Our data also provide some clues to possible evolutionary connections between Vinitor and (pro)phages associated to epiphytic and insect-related bacteria.

## Introduction

Lactic acid bacteria (LAB) are among the most important groups of microorganisms used in food fermentations (Bintsis, 2018). Sustained consideration has been given to their cognate bacteriophages which traditionally cause fermentation failure and product inconstancies. The largest and best documented problems originated by phage presence have been described in dairy industries, leading to substantial economic losses (Pujato et al., 2019). This has driven extensive diversity and ecological studies of LAB-infecting phages within dairy environments, resulting in some improvement of the phage-resistance of starter cultures (Mahony and van Sinderen, 2015; McDonnell et al., 2018). The vast amount of information produced also yielded valuable insights into the mechanism which govern phage recognition and penetration into Gram positive bacteria (Dunne et al., 2018; Martínez et al., 2020), and provided many key advances that now define our understanding of the complex evolutionary relationships between tailed phages (Kupczok et al., 2018). Hence, data progressively put forward the notion that some LAB phages could inherit structural genetic modules from various phages of dairy as well as non-dairy species (Mikkonen and Alatossava, 1995; Labrie et al., 2008; Samson and Moineau, 2010; Szymczak et al., 2017; Philippe et al., 2020). Such connections between phages infecting different species can now be nicely captured by genome-level network analyses (Iranzo et al., 2016; Shapiro and Puntonti, 2018). Using this perspective, the analysis of phages which have no genes in common, can show the existence of a set of possible paths that can connect each of their genes in relatively few steps (Dion et al., 2020). Such a holistic approach is developing, and continued efforts are now needed to better characterize the globally connected phage gene pool and increase their connectivity. Adding more LAB phage sequences from phylogenetically related hosts to reference databases may be helpful (Dion et al., 2020) and will require investigations of other types of fermented foods such as meat, cereals, vegetable and fruits (Kot et al., 2014; Chukeatirote et al., 2018).

Wine-associated communities may represent promising candidates for such exploration, as they include various LAB genera belonging to the emended family *Lactobacillaceae* (Zheng et al., 2020) such as *Leuconostoc*, *Fructobacillus*, *Oenococcus*, and *Weissella*, whose infection by phages is so far poorly documented (Kot et al., 2014). The enological environment is complex and characterized by temporal succession of distinct communities of microorganisms, with highly interconnected networks of metabolic and ecological interactions with other niches such as plants and their rhizosphere, soils, insects, and humans (Morgan et al., 2017; Morrison-Whittle and Goddard, 2018). The hypothesis that wine-associated communities may turn out to represent a valuable source for hitherto undescribed phages is supported by the recent description of novel genera of phages infecting *L. plantarum* (Kyrkou et al., 2019, 2020), a LAB capable of performing the malolactic fermentation (MLF) in high pH wine (Krieger-Weber et al., 2020). The latter process, which reduces acidity and increases microbial stability, creates good quality grape wine, and is important for the aging of red wines and certain white wines.

Current projects of our group focus on phage-host interaction in the other and uncontested queen of the cellar, namely *Oenococcus oeni* (Grandvalet, 2017). This LAB species is better adapted to the limiting conditions imposed by the wine matrix and performs the crucial role of MLF under regular winemaking conditions, especially in wines with a pH of below 3.5. Similar to other food fermentations, cases of stuck and sluggish fermentations are annually reported worldwide. They lead to depreciation of product quality, have negative economic impact and are difficult to manage in the wine industry. Commercial MLF starter cultures which mostly consist of strains from the *O. oeni* species have been selected. However, their use does not yet ensure a problem-free fermentation. Difficulties arise from a combination of factors, including the presence of phages infecting *O. oeni* (oenophages) (Malherbe et al., 2007). We have previously isolated and characterized various oenophages which originated from distinct wine fermentation samples, and a range of geographical locations and time points (Jaomanjaka et al., 2016; Philippe et al., 2017). Most corresponded to temperate or ex-temperate siphophages suggesting that bacterial strains are the main reservoir for oenophages (Jaomanjaka et al., 2016; Philippe et al., 2017). Accordingly, *in silico* analyses showed that *O. oeni* genomes are replete with integrated prophages (Bon et al., 2009; Borneman et al., 2012; Jaomanjaka et al., 2013). Noteworthy, lysogeny was shown to be prevalent among MLF commercial cultures and further studies are now needed to better undertand lysis-lysogeny decision strategies in wine. Intringuinly, a small set of oenophages collected in 2015 drew our attention as they produced clear plaques and did not exhibit several key genomic features which are globally applicable to previously described temperate oenophages (Philippe et al., 2017; Chaïb et al., 2019). In the current study, whole-genome sequencing of two phages of the group, further named Vinitor, confirmed their virulent nature, reinforcing the notion that phage predation of *O. oeni* occurs during wine making. Despite absence of homology at the DNA level with other fully sequenced phages, several Vinitor-encoded putative proteins showed sequence similarity to proteins of dairy LAB phages. Our data also provide somes clues to possible evolutionary connections between Vinitor and (pro)phages associated to epiphytic and insect-related bacteria.

## Materials and Methods

### Propagation of Vinitor Phages

Lysates of Vinitor162 or Vinitor27 were produced on *O. oeni* IOEB-SARCO 277 which does not contain endogenous phage and is sensitive to all oenophages so far isolated in our laboratory (Chaïb et al., 2019). Phage amplification was carried out in MRS broth supplemented with MgSO_4_ (3.75 g/L) and CaCl_2_ (2.375 g/L) (MRS_Φ_) with a multiplicity of infection of 0.03. After 3 days, the lysed culture was centrifuged and the supernatant was filtered through a 0.2 μm polyethersulfone (PES) membrane filter (Jaomanjaka et al., 2013). The lysate was titered on the same host using the classical double-layer plating technique. MRS_Φ_ agar plates were placed in unsealed plastic bags and incubation was carried out at 25°C for 4–7 days to allow plaque formation.

Each lysate was subsequently typed using four PCR tests that distinguish the Int_*A*_, Int_*B*_, Int_*C*_, and Int_*D*_ groups among oenophages, based on their integrase (*int*) sequence (Jaomanjaka et al., 2013; Philippe et al., 2017). Alternately, phage particles were replaced with 0.5 μL of template DNA (50 ng). A Biorad i-Cycler was used for the amplification reactions, which were achieved in a 25 μL volume using the Bio-Rad Taq PCR Master Mix kit and 0.2 μmol/L of each primer. The phage DNA released by heat lysing of particles (10^3^ PFU per reaction) served as template for PCR. All oligonucleotides were purchased from Eurofins MWG-Operon (Munich, Germany).

### Incidence of Vinitor Phages During the 2015 Vintage

We explored the presence of oenophages along 23 red and white wine fermentations in 7 wineries during the 2015 vintage (Supplementary Table 1). Most were sampled at different steps of the process (must, alcoholic fermentation (AF), MLF and post-blending) yielding a set of 127 samples. Two distinct protocols were used as previously described (Philippe et al., 2017). Briefly, in protocol 1, samples were centrifuged (5,000 g, 10 min) and filtered on 0.2 μm membrane filters made of polyether sulfone. In protocol 2, samples were 10-fold diluted in MRS liquid medium supplemented with 0.1 mg/mL pimaricin, incubated for 5 days at 25°C, centrifuged and filtered as described above. All samples were stored at 4°C.

Phages present in the samples were titered using the classical double-layer plating technique as described above. Following incubation, single plaques were picked, suspended in 0.5 mL of sterile MRS_Φ_ medium and stored at 4°C. They were propagated on the same strain and reisolated two more times to ensure the purity of the phage lysates. To obtain high-titers phage lysates, 10 confluent lysis plates were prepared. Soft agars were collected, centrifuged, and the supernatant was filtered. Phage titers ranging from 10^8^ to 10^9^ PFU/mL were obtained.

Phages recovered from the vintage 2015 were tested using the integrase PCR tests. The lysates with no amplification signals were considered as putative Vinitor phages. They were subsequently typed using primer couples designed in five genes from Vinitor162 which specify the Terminase large sub-unit, Major Capsid protein, Tail length tape-measure, Putative Tal and phage replisome organizer (Table 1). Primer design was achieved by using the eprimer3 0.4.0 and Oligo analyser 1.0.3 software.

**TABLE 1:** Primers used in this study.

**ORF**	**Putative function**	**Sequence (5′->3′)**	**Size of the Amplicon (bp)**
Gp2 Vinitor162-f	Terminase large sub-unit	ccgtgcccgtttatcaagag	157
Gp2 Vinitor162-r		tacactctaccttgcgccaa	
Gp8 Vinitor162_f	Major Capsid protein	agcaaacgatggggcaaatt	363
Gp8 Vinitor162_r		gaggtgcgtcatgttcagac	
Gp16 Vinitor162-f	Tail length tape-measure protein	caaaatatggcgcttcggga	207
Gp16 Vinitor162-r		tttctgcacttgtcccgttg	
Gp18 Vinitor162-f	Tal protein	tgccaactgaagcaaatccc	460
Gp18 Vinitor162-r		gcattactccacgctgtctg	
Gp30 Vinitor162-f	Phage replisome organizer	ggcaagcctgacaattcatca	396
Gp30 Vinitor162-r		taatgcatgctccgcttcac	

### Host Spectra

The propagation of phage Vinitor162 was tested on a panel of *O. oeni* strains and other LAB (Table 2 and Supplementary Table 2). The resistance level of bacterial strains to phages was expressed using the efficiency of plating (EOP) ratio. The EOP was defined as the ratio between PFU/mL obtained on each putative resistant strain and PFU/mL obtained on the strain initially used for the phage propagation (*O. oeni* IOEB S277). Resistant strains were represented by EOP values < 1.

**TABLE 2:** Host range analysis of phage Vinitor162 against *Oenococcus oeni* strains and other lactic acid bacteria.

**Species**	**Name**	**Origin/commercial name**	**Phylogroup**	**Prophage type**	**EOP**
*O. oeni*	IOEB C52	Cider, Normandy, F	C	Int_*A*_	<10^–8^
	Sarco S13	Red wine, F	B	Int_*D*_	<10^–8^
	IOEB C23	Cider, Normandy, F	B	Int_*B*_	<10^–8^
	IOEB C28	Cider, Brittany, F	B	Int_*D*_	<10^–8^
	IOEB 9803	Red wine, F	B	Int_*D*_	<10^–8^
	IOEB 9805	Red wine, F	B	Int_*D*_	<10^–8^
	AWRI B429	Lalvin VP41 Lallemand	A	Int_*A*_ and IntD	10^–5^
	IOEB 0608	Wine, F	A	Int_*A*_	9.8 × 10^–4^
	Sarco S11	Sparkling white wine, F	A	Int_*D*_	<10^–8^
	IOEB CiNe	Starter CHR Hansen	A	Int_*A*_ and Int_*C*_	10^–2^
	Sarco 28	B28 PreAc, Laffort, F	A	Int_*A*_ and Int_*C*_	<10^–8^
	IOEB 9517	Floc de Gascogne, F	A	None	7.8 × 10^–4^
	IOEB S450	450 PreAc Laffort, F	A	None	<10^–8^
	IOEB 1491	Red wine, F	A	Int_*A*_	10^–1^
	S19	Red wine, F	A	Int_*A*_ and Int_*C*_	1
	IOEB B10	Red wine, F	A	Int_*B*_	1
	IOEBS277	Red wine, F	A	None	1
	S15	Red wine, France	A	None	1
	S161	350 PreAc Laffort, France	A	Int_*A*_	1
	S25	Red wine	A	None	<10^–8^
	IOEB L26.1		A	Int_*A*_	1
	S26		A	None	1
*O. kitaharae*	NRIC_0647	Shochu residue, Japan	ND		<10^–8^
	NRIC_0649	Shochu residue, Japan	ND		<10^–8^
	NRIC_0650	Shochu residue, Japan	ND		<10^–8^
*O. alcoholitolerans*	JP736	Ethanol production	ND		<10^–8^
	M7212	Ethanol production	ND		<10^–8^
*L. plantarum*	CRBO AL2	Enological environment	ND		<10^–8^
	CRBO AL210	Enological environment	ND		<10^–8^
	CRBO ERS1.11	Enological environment	ND		<10^–8^
	lp80		ND		<10^–8^
	BMS2	Buccal environment	ND		
*P. pentosaceus*	ICP01		ND		<10^–8^
	NRRC		ND		<10^–8^
	ATCC 33316		ND		<10^–8^

### Genome Sequencing

The phage lysate was concentrated by ultracentrifugation and double-stranded DNA was extracted as described previously (Chaïb et al., 2020). Whole-genome sequencing was performed at the Genome-Transcriptome facility of Bordeaux^1^. DNA libraries were prepared using the Nextera XT DNA library preparation kit (Illumina, San Diego, CA). Genomic DNA was sequenced using an Illumina MiSeq using 2 × 250 bp paired-end libraries. Reads were assembled using SPAdes (Bankevich et al., 2012) with default parameters (read correction and assembler). The assembly of the whole-genome sequences was verified using *Hin*dIII-restriction profiles of phage DNAs and gel electrophoresis.

The full genome of *O. oeni* phage Vinitor162 and Vinitor27 were deposited in GenBank under the accession numbers MF939898 and MT859305.

### Genomic Comparisons and Phylogenetic Analyses

The *in silico*-translated protein sequences were used as queries to search for sequence homologs in the non-redundant protein database at the National Centre for Biotechnology Information using BLASTP (Altschul et al., 1997) with an upper threshold E-value of 1 × 10^–3^.

Searches for distant homologs were performed using HHpred (Söding et al., 2005) against different protein databases, including PFAM (Database of Protein Families), PDB (Protein Data Bank), CDD (Conserved Domains Database), and COG (Clusters of Orthologous Groups), which are accessible via the HHpred website. Searches against the CDD database at NCBI were also performed using CD-search (Marchler-Bauer and Bryant, 2004).

For gene phylogeny analyses, sequences were aligned using ClustalOmega. Maximum likelihood phylogenetic trees were constructed using the PhyML (Guindon et al., 2010), the latest version of which includes automatic selection of the best-fit substitution model for a given alignment. A Bayesian-like transformation of aLRT (aBayes), as implemented in PhyML (Guindon et al., 2010) was used to estimate branch support. For genomes, all pairwise comparisons of the nucleotide sequences were conducted using the Genome-BLAST Distance Phylogeny (GBDP) method (Meier-Kolthoff et al., 2013) using VICTOR under settings recommended for prokaryotic viruses (Meier-Kolthoff and Göker, 2017). The resulting intergenomic distances were used to infer a balanced minimum evolution tree with branch support via FASTME including SPR postprocessing (Lefort et al., 2015) for each of the formulas D0, D4, and D6, respectively. Branch support was inferred from 100 pseudo-bootstrap replicates each. Trees were rooted at the midpoint (Farris, 1972) and visualized with FigTree 1.4.3 (Rambaut, 2006). Taxon boundaries at the species, genus and family level were estimated with the OPTSIL program (Göker et al., 2009), the recommended clustering thresholds (Meier-Kolthoff and Göker, 2017) and an *F*-value (fraction of links required for cluster fusion) of 0.5 (Meier-Kolthoff et al., 2014). Then the best yielding average supported tree was exported with the Newick tree format and design to iTol 5.6.1 (Letunic and Bork, 2019).

GenBank accession numbers of phage genomes and names of infected species used in the phylogenetic analysis are the following: phiLN03 (KC013022), Lmd1 (JQ659259.1), P793 (KC013021.1) (*Leuconostoc pseudomesenteroides*); phiLN34 (KC013027.1), phiLN25 (KC013021) (*Leuconostoc mesenteroides*); Ld3 (KJ564038.1), c5 (EU340421.2), Ldl1 (KM514685.1), ViSo-2018a (CP031026.1), LL-H (EF455602) (*Lactobacillus delbrueckii*); Silenus (MG765278.2), P1 (KX223815.1), Maenad (MG765274.1), ATCC8014-B2 (JX486088), Nyseid (MG765276.1), Lenus (MG252693.1), phig1e (X98106) (*Lactiplantibacillus plantarum*); S-AC12 (KU052488) (*Levilactobacillus brevis*)*;* phiAT3 (AY605066) *Lacticaseibacillus casei*; phage ATCC 27139 (PL-1) (KC171647) (*Lacticaseibacillus paracasei*); LF1 (HQ141410) (*Limosilactobacillus fermentum*); Lj965 (AY459535), Lj928 (AY459533), Lj771 (NC_010179) (*Lactobacillus johnsonii*); OE33PA (MH220877.1) (*O. oeni*); DT1 (AF085222.2), 7201 (AF145054.1), O1205 (U88974.1), 5093 (FJ965538.1), 9871 (KU678389) (*Streptococcus thermophilus*); TP901 1 (AF304433.1), BK5-T (NC_002796), bIL285 (AF323668.1), r1t (U38906.1), KSY1 (DQ535032.1), P738 (MK911750), 109751 (MK044829.1), bIL312 (AF323673.1), sk1 (AF011378), c2 (L48605.1), P087 (FJ429185.1), 1706 (EU081845.1), 1358 (GQ403788) (*Lactococcus lactis*); P35 (DQ003641.1) (*Listeria monocytogenes*). Prophages were obtained from the following lysogens *O. oeni* B10 (NZ_AZJW00000000), *O. oeni* IOEB0608 (AZKJ00000000), and IOEBS1491 (AZLG00000000); *Liquorilactobacillus vini* DSM20605 (AHYZ00000000), *Ln. mesenteroides* NRRL-B512F (CP046062.1), and LBAE-G15 (AMD77912.1); *Limosilactobacillus mucosae* CRL573 (JROC00000000); and metagenomic data from *Convivina intestini* (QEKT00000000).

## Results and Discussion

### Vinitor162 Is a True Virulent Oenophage

**FIGURE 1:**
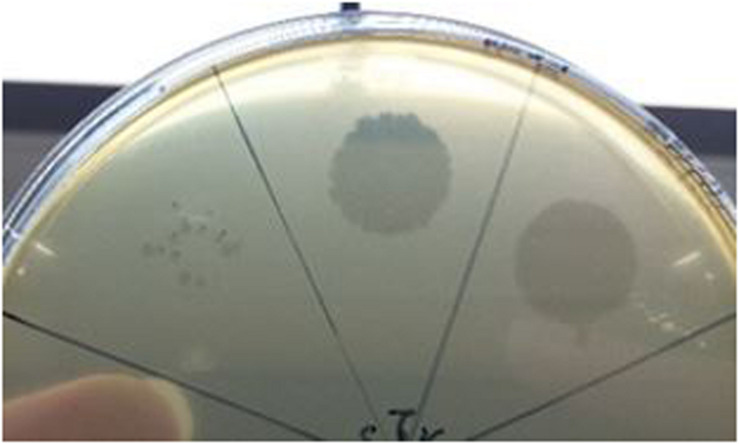
Plaque morphology of phage Vinitor162 on its *O. oeni* host.

**FIGURE 2:**
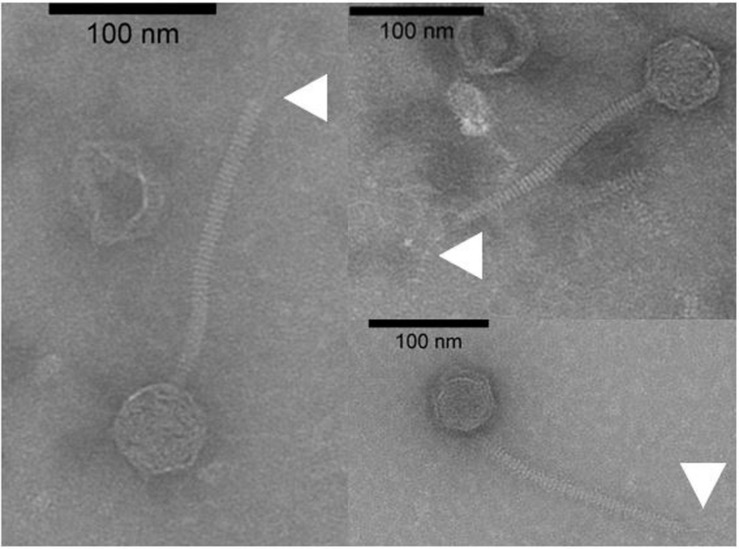
Uranyl acetate-stained transmission electron micrographs of phage Vinitor162. The white arrows show the extended unique thin tail fiber.

To date, limited studies of *O. oeni* phages have been undertaken despite the commercial relevance of this bacterial species during winemaking. The few available studies have mostly focused on the characterization of mitomycin C-induced temperate oenophages, showing a high incidence of lysogeny in the species (Jaomanjaka et al., 2013). The lack of isolated virulent (non-temperate) oenophages, although intringuing, was interpreted as an indication of an insufficiently sampled environment. As from 2013, we decided to explore the whole oenological reservoir for this specific phage hunt. Our strategy was to sample all wine-types and essential steps (must, AF, MLF, aging) of the winemaking process and to achieve a faster processing of the samples as possible. As a first result, a set of 31 putative virulent phages of *O. oeni* were succesfully isolated from the 2014 vintage (Philippe et al., 2017). Briefly, these phages exhibited unusual genomic characteristics, of which the lack of the integrase and endolysin genes that are the signature of temperate oenophages. They were initially called unk (for unknown) and renamed Vinitor (latin name for winemaker) in our laboratory to reflect their origin. Vinitor162 phage isolated from white wine (Sauternes, France) was arbitrarily chosen for further characterization. It was propagated on *O. oeni* IOEBS277 to titres of 10^8^ to 10^9^ PFU/mL. Both the clear phenotype and small size of the plaques observed earlier were confirmed (Figure 1). Concentrated phages were recovered as previously reported (Chaïb et al., 2020) and TEM observations showed that phage Vinitor162 belongs to the *Siphoviridae* family, with an icosahedral head (55 ± 3 nm in diameter, *n* = 20), and a non-contractile tail (205 ± 8 nm, *n* = 20) with an extended unique thin tail fiber at its extremity (Figure 2).

To determine the host range of Vinitor162, 22 representative *O. oeni* strains were chosen from the CRBO collection, based on their assignment to one of the three main phylogroups (A, B, and C) described in the species (Lorentzen and Lucas, 2019) and their distinct prophage contents (Table 2). The host range test indicated that Vinitor162 is able to efficiently infect 12 different *O. oeni* strains, all from phylogroup A, including some commercial MLF starters. Intermediate EOPs (10^–2^ to 10^–5^) were observed against 4 strains (AWRIB429, IOEB0608, IOEBCine, IOEB9517), suggesting impaired adsorption or the presence of a resistance mechanism against Vinitor162. Eight out of the 12 sensitive strains were mono- or poly-lysogens and resident prophages belonged to so far identified Int_*A*_, Int_*B*_, Int_*C*_ or Int_*D*_ groups (Jaomanjaka et al., 2013). Those temperate phages therefore did not exhibit immunity to superinfection by phage Vinitor162 (Table 2). Last, Vinitor162 could not form plaques on any of the tested strains belonging to other species of the genera *Oenococcus*, *Pediococcus* and *Lactiplantibacillus, Lactobacillus, Lacticaseibacillus*, and *Limosilactobacillus* isolated from the enological environment and beyond (Table 2 and Supplementary Table 2).

### Genomic Characteristics of Phage Vinitor162

**FIGURE 3:**
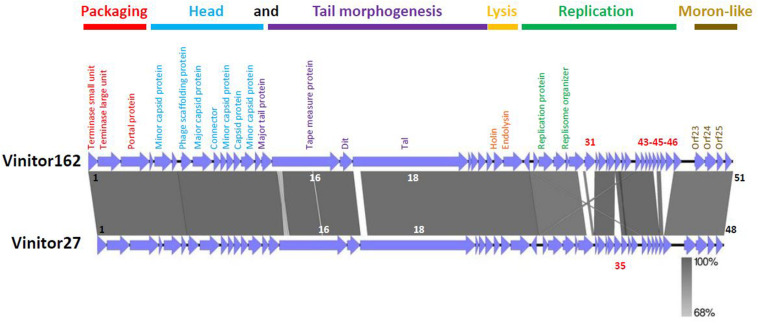
Architecture of the oenophages Vinitor162 and Vinitor27 genomes. The predicted functions of the Vinitor genes are indicated below the maps. The presumptive modules are colored. The gray vertical blocks between phage sequences indicate regions of shared homology according to BLASTn, and the degree of nucleotide identity is indicated by the intensity of gray. ORFs in white characters have small differences in their nucleic sequences and those in red are specific to each Vinitor phage. The figure was generated with Easyfig.

The phage genome was sequenced using the Illumina MiSeq platform, resulting in a complete genome sequence with a length of 36,299 bp, and an average coverage of 2869 X. The G+C content was 36.14%, which is close to that of the bacterial host (37.9%). No matches to any other entity were found by BLAST searching at the nucleotide level. The genome had 51 predicted *orfs* and contained no *tRNA* genes. The smallest gene preceded by an adequate ribosome binding site (RBS) would encode a protein of only 49 amino acids (gp34). The sizes of the remaining gene products varied from 55 (gp32) to 2,170 amino acids (gp18). All *orfs*, but *orf*25, were positioned on the same DNA strand. The BLAST program was used to compare the protein sequences from phage Vinitor162 to sequence databases, and the salient genome characteristics are outlined in Supplementary Table 3, with a detailed list of top BLAST identities. A large proportion of the predicted gene products (31 of 51, 61%) showed no obvious predicted biological function. The majority of them (18 of 31) had no matches in the databases, reinforcing the idea that Vinitor162 presents high levels of divergence from known oenophage genomes, and confirming that the LAB phage pool is still largely unexplored. Orphans were not randomly distributed on the genome and a total of 14 *orfs* were located in two specific regions (*orf*19-*orf*22; *orf*34-*orf*47) (Figure 3). Protein homology was detected for 33 of the 51 deduced proteins and identity levels were ranging from 29 to 95%. Of note, a vast majority of homologs (*n* = 29) were clearly present in prophages (usually unannotated), suggesting their role as a possible sequence reservoir. Half of these Best BLAST hits (BBH) results (*n* = 15; 29.4% of total ORFs) were related to prophages/phages infecting species of the *Oenococcus* genus (*O. oeni* and *O. sicerae)* or closely related taxa such as *Weisella* sp., and lactiplantibacilli such as *L. plantarum*, which tend to share similar ecological niches with *O. oeni*.

### Architecture of the Genome of Vinitor162

The architecture of the phage genome is shown in Figure 3. We assigned the bp +1 to the first base pair of the predicted small terminase subunit gene, so that its map can be easily compared to other phages of LAB. Of the 51 predicted genes, functions could only be predicted for 20 of the putative proteins they encode, and the majority of these were phage structural proteins (see below). The phage did not encode recognizable integrase, excisionase or repressor genes confirming that Vinitor phages have an exclusively lytic life cycle. Despite the peculiarity of its genome sequence and high number of orphans, phage Vinitor162 shared synteny with other reported temperate/ex-temperate oenophages in terms of genome organization, with the DNA packaging module followed by the structural module, the lysis module and the replication module (Jaomanjaka et al., 2013; Chaïb et al., 2019).

#### Packaging Mechanism and Genome Extremities

**FIGURE 4:**
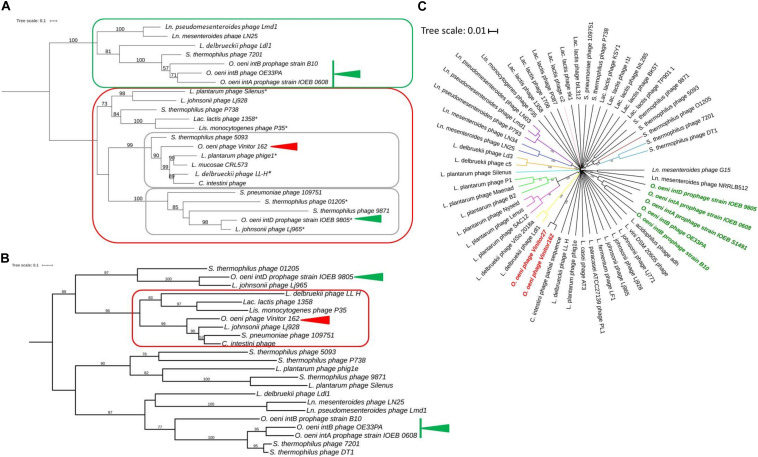
Phylogenetic relationships of Vinitor phages and related phages. Phylogenetic trees using the TerL **(A)**, MCP **(B)** proteins and whole genome nucleotide sequences **(C)** are shown. A and B; Maximum likelihood phylogenetic trees were computed using the best-fit substitution models for given alignments as determined by PhyML with Bayesian Information Criterion with a LRT SH-like method for branch support as reported above. The values are shown for nodes with ≥ 50% support. The scale bar represents the number of amino acid substitutions per site. Asterisks represent *pac*-containing phages. Red and green arrows represent Vinitor162 and other oenophages, respectively. C. Phylogenetic position of Vinitor162 and Vinitor27 using VICTOR. Current virus taxon nomenclature by ICTV is given with a color code for the different branches as follows: sub-family *Mccleskeyvirinae* Genus *Limdunavirus* (purple); sub-family *Mccleyskyvirinae* Genus *Unaquatrovirus* (blue); Genus *Cequinquevirus* (orange); Genus *Coetzeevirus* turquoise blue); Genus *Maenadvirus* (bright green); Genus *Lenusvirus* (fuschia); sub-family *Tybeckvirinae* Genus *Lidleunavirus* (yellow); Genus *Brüssowvirus* (brown); Genus *Moineauvirus* (light blue); Genus *Ceduovirus* (pink); Unclassified virus (black); Vinitor phage (bold red); other Oenophages (bold green).

We did not obtain experimental evidence of the presence of cohesive genomic extremities in Vinitor162 during our previous work (Philippe et al., 2017). Analysis of the raw Illumina reads by PhageTerm (Garneau et al., 2017) also ruled out their presence in the phage genome, but no packaging mechanism could be further assigned to phage Vinitor162. This situation has been also reported for other phages such as the *Clostridium* phage phiCD11 (Garneau et al., 2017) and the *Streptococcus* Str01 and Str03 phages (Harhala et al., 2018) and could be a result of Nextera library preparation method. The reconstructed phylogeny based on amino acid sequence of phage terminase large subunits (TerL) was recently shown to produce clusters associated with types of genome terminus and encapsidation mechanism (Clokie and Kropinski, 2009). We analyzed a data set of TerL sequences from phage Vinitor, as well as representatives of the inducible prophages belonging to the Int_*A*_, Int_*B*_, and Int_*D*_ groups in *O. oeni*, and other phages of LAB present in public databases (Figure 4A). TerL sequences segregated phages into 2 well-supported clusters. We first observed a joint branching of the *cos*-containing oenophages, namely the Int_*A*_ and Int_*B*_ oenophages (Gindreau et al., 1997; Jaomanjaka et al., 2013, 2016) with some well characterized LAB phages such as Ldl1 from *L. delbrueckii* (Casey et al., 2015) and phage 7201 infecting *S. thermophilus* (Stanley et al., 1997), which have similar genome extremities. In contrast, both the Int_*D*_ prophage and virulent phage Vinitor belong to a second cluster, which harbors many terminally redundant and circularly permuted phages due to headful packaging (*pac* phages) (Figure 4A). Of note, the temperate Int_*D*_ prophage is predicted to be a *pac*-type by Phage Term (data not shown). It can therefore be inferred that phage Vinitor may have headful packaging.

#### The Morphogenesis Module

The genes immediately downstream of *orf2* encode a set of structural proteins which are presumed to be involved in phage head assembly. *gp3* encodes the portal protein, which assembles in a multi-subunit annular structure at one corner of the icosahedral shell, where it serves as the entrance and exit door for the viral DNA, as the site for head assembly, and the attachment site for the tail (Dedeo et al., 2019). BLASTP searches showed significant sequence homology with the portal protein of uncharacterized prophages found in *L. sakei*, and various phages infecting pathogenic *Streptococcus* species such as *S. pneumoniae* (Croucher et al., 2016) and *S. gordonii* (Javan phages) (36% identity and e-values of 1e-100) (Rezaei Javan et al., 2019; Supplementary Table 3). The module also comprised other putative structural proteins (gp5 to gp13) of which gp7 may represent the capsid scaffolding protein, gp8 the major capsid protein (MCP), gp9 the connector and gp13 the major tail protein.

The structural components of the bacteriophage tail resembled the complex system found in a majority of characterized LAB phages, comprising a triad of proteins (gp16, tape measure protein, TMP; gp17, distal tail protein, Dit and gp18, tail associated protein, Tal) which allow the recognition and binding to host cell surface-located polysaccharides, or more rarely proteins (Goulet et al., 2020). Gp16 was assigned to the TMP, which determines the length of the tail and participates in the infection mechanism. The length of the siphophage tail is directly proportional to the size of the TMP, where one amino acid of the TMP equals 0.145 nm of tail length (Mahony et al., 2016). Using this equation, the length of the tail in Vinitor162 was expected to be 185 nm (TMP, 1,274 amino acids), which correlated well with our estimation from the electron micrographs (Figure 2). Downstream, the first gene encountered in Vinitor162 was that of the distal tail protein (Dit), gp17. Hexameric Dit proteins are conserved in *Siphoviridae* infecting LAB and are attached to the last tail hexamer MTP (major tail protein) (Dunne et al., 2018). Of note, Dit from Vinitor162 is 243 aa long and falls within the range of reported sizes for classical-length Dits (∼260–300 amino-acids) (Dieterle et al., 2017; Hayes et al., 2018). On its other side, the Dit protein is known to bind to a trimeric protein named Tal (tail associated lysin). The latter belongs to the vision-associated peptidoglycan hydrolases (VAPGHs) and their main function is the peptidoglycan layer degradation, to facilitate phage adsorption. Recent progress in LAB phage research illustrates that Tal can represent the most complex and heterogeneous elements of the virions (Lavelle et al., 2018). As for Vinitor162, we found that the Tal protein (gp18) was very long (2,170 aa), comparable to the length of Tals of *cos* and *pac*-containing phages from *Streptococcus thermophilus* (now *Moineauvirus* and *Brüssowvirus*) genera (Lavelle et al., 2020). This *orf* was indeed the longest of the Vinitor162 genome.

#### Other Predicted Proteins

The gene product of *orf24* has the hallmarks of an endolysin and contains a muramidase domain. It is highly homologous to several LAB phage endolysins, of which that of a prophage of*O. sicerae*, a species found in ciders (Cousin et al., 2019). Further sequence analysis by HHpred revealed that a large part of gp23 (residues 1–109) was predicted as a holin domain associated to phage LL-H (27% identity). Hence, as usually observed amongst siphophage genomes, the holin gene directly precedes the endolysin gene.

Downstream the lysis module, the phage genome contained a large region of about 23 short *orfs* (*orf25*-*orf47*) encoding 12 proteins with no significant hits to any sequences in GenBank. The proteins deduced from 6 *orfs* had homologs in *O. oeni* or *O. sicerae*, but no function or conserved domain was identified. Only four proteins had significant homology to proteins involved in replication: gp27, a replication protein; gp30, a putative phage replisome organizer and gp33, a YopX-like protein found in the replication module of a variety of phages. Corresponding BBH were associated with the *Enterococcus* phage phiEf11 (29% identity) and prophage sequences from *W. fabalis* (50% identity) and *O. oeni* (74% identity). Of note, gp31 was highly related to a protein associated with the ex-temperate oenophage OE33PA (61% identity).

Strikingly, *orfs 48-49-50* reassembled 3 of the 4 moron genes also found upstream of *terS* in the LAB temperate phage P335 and transcribed autonomously in the *Lac. lactis* host (Labrie et al., 2008). The moron sequences were not found in the other characterized lactophages of the P335 group. Yet homologous sequences were detected in the genomes of *Ent. faecalis*, *S. pyogenes*, and *L. innocua* prophages. Our data broaden the network of phages sharing this specific module of genes, and also confirms the hypothesis of a specific role in providing fitness factors to phages.

### Diversity in the Vinitor Group and Relevance of Members During Winemaking

In an attempt to assess the incidence and diversity of Vinitor phages in the enological environment, we performed an additional phage survey during the 2015 vintage. The study was not designed to be an epidemiologically representative survey of phage distributions, so prevalences are not meaningful, but some observations can be made. We explored the presence of oenophages along in 127 samples, and 64 pure lysates were produced (Supplementary Table 1). Based on PCR tests with *int*-specific primers, a total of 58 newly isolated phages were classified in one of the four distinct groups of temperate oenophages (Supplementary Table 1). The 6 remaining phages which did not contain any of the identifiable sequences conserved in the integrase of oenophages formed clear plaques on the sensitive host IOEBS277. Presence of presumptive virulent oenophages during two successive years suggests that such phages are components of the phage community associated to winemaking.

Next, we designed novel Vinitor-specific PCR assays to further explore the diversity of the set of virulent phages isolated during the 2014 (*n* = 31) and 2015 (*n* = 6) vintages. They targeted five genes in the genome of Vinitor162: *terL* (*orf*2), *mcp* (*orf*8), *tmp* (*orf*16), *tal* (*orf*18), and *rep* (*orf*27) sequences (Table 1). A total of 35 phage DNAs were tested positive and yielded the expected amplicons in the five PCR assays. A single phage, namely Vinitor27, isolated from a red wine (Merlot) exhibited slightly different results as no amplicon were produced with the primers designed in the *rep* gene, suggesting variability in this region of the chromosome. The genome of Vinitor27 was sequenced and revealed little variation in genome size and number of *orfs*. It was 35,279 bp in length, with 48 predicted genes (Figure 2). Both Vinitor phage genomes shared a 97.6% identity at the nucleotide level and should therefore be considered as the same species. Genomic synteny tests with Easyfig visualized the previously manifested homology among the two Vinitor phages and provided evidence for their conserved genome architecture, with 47 common genes. Of note, the poorly characterized replication module was affected by some insertion/deletion events, explaining the differences observed earlier in the PCR-based assays. As a consequence, four small orphans in the replication module (*orf31*, *orf43*, *orf*45, *orf*46) were found only in Vinitor162, while *orf35* (also an orphan gene) was specific to Vinitor27 (Figure 2).

An interesting question was to assess whether virulent phages have specific temporal patterns of abundance during winemaking compared to temperate oenophages. We reexamined the 83 positive samples for the presence of phages which were collected during the 2014 and 2015 vintages. Vinitor phages were prevalent in all types of wines (20 phages were isolated from red wines and 16 from dry and sweet white wines). We next analyzed the abundance of the distinct groups of oenophages at the different steps of the winemaking process (must, AF, MLF and aging) (Table 3). Ten out of 16 samples of musts contained a Vinitor phage (62.5%). The frequencies were reduced in samples collected during AF (21.7%), MLF (22.2%) and aging (50%). The results obtained with «aging» samples should, however, be treated with caution as only 8 samples were analyzed. In comparison, about 95% of the phage-containing samples originating from AF and MLF contained temperate oenophages, while their frequency was reduced to 37.5% in must samples (Table 3). Last, Vinitor27 was isolated from a sampling time serie, allowing us to catch a glimpse of its persistence during the fermentation of Merlot grapes (Entre-Deux Mers, France). The virulent phage was detected in must (2 × 10^2^ PFU/mL) and not in any further steps (AF, MLF). Of note, Int_*A*_ oenophages were detected later, during MLF. Taken together, these preliminary data suggest that a temporal succession of distinct oenophages occurs throughout the winemaking process. Vinitor phages may be transiently active during the early stage of winemaking when grapes are crushed, while temperate phages are more prevalent in subsequent steps, probably upon excision from indigenous lysogenic strains of *O. oeni.*

**TABLE 3:** Prevalence of Vinitor phages during two isolation campaigns (2014 and 2015).

**Step**	**Positive sample^*a*^**	**Type of oenophages isolated from positive samples and prevalence (%)**								
		**Temperate only**					**Vinitor only**	**Vinitor and temperate**	**% with temperate**	**% with Vinitor**
		**Int_*A*_**	**Int_*B*_**	**Int_*C*_**	**Int_*D*_**	**Mixed**				
Must	16	2	1	–	3	–	10	0	37.5	62.5
AF	23	6	11	–	–	1 (Int_*A*_+Int_*B*_)	1	4 (Vinitor+Int_*A*_)	95.6	21.7
MLF	36	14	10	–	–	1 (Int_*A*_+Int_*B*_) 3 (Int_*B*_+Int_C_*)	2	5 (Vinitor+Int_*A*_) 1 (Vinitor+Int_*A*_+Int_*D*_)	94.4	22.2
Aging	8	3	1	–	–	–	1	2 (Vinitor+Int_*A*_) 1 (Vinitor+Int_*A*_+Int_*D*_)	87.5	50

^*a*^33 and 50 samples containing phages were collected in 2014 and 2015, respectively. *Pure Int_*C*_ phages could not be isolated.

### Topology Studies Predict a Peculiar Adhesion Apparatus of Vinitor Phages

With the aim of getting some structural information on the adhesion device of Vinitor phages, we performed HHpred (Söding et al., 2005) analysis of the *orfs* comprised between the TMP and the Lysin (TMP+8). HHpred of Dit (TMP+1) confirmed that it is a classical Dit, with the topology of phage SPP1 Dit (PDB ID 2X8K, probability 100%, Veesler et al., 2010). Tal (TMP+2) analysis yielded several hits (Supplementary Figure 1). Contrary to most cases, the classical N-terminal structural domain found in all Tals (Goulet et al., 2020) is identified by HHpred not at the N-terminus, but between residues 156 and 505 (PDB ID 3GS9, *Listerial* phage protein). Preceeding it, the domain comprised between amino-acids 1 and 155 in Vinitor162 is identified as Carbohydrate Binding Module 1 (CBM), a BppA (base plate protein) domain of lactococcal phage Tuc2009, a module widely spread in siphophages infecting Gram-positive hosts (PDB ID 5E7T, Legrand et al., 2016). Noteworthy, Tal sequences of both Vinitor phages differ largely within the 141 first residues corresponding to this predicted module (Figure 3 and Supplementary Figure 1) while they share ∼98% identity between residues 142 and 2,170. This is suggesting that the domain represents a variable region amongst Vinitor phages. The biological significance of this difference is currently unclear. Following residue 505, three CBMs (CBMs 2–4) were identified by HHpred at positions 784-929, 1,182–1,329, and 1,640–1,788, separated by linkers, along the extended Tal (Supplementary Figures 2, 3). Finally, a fifth CBM domain (CBM5) was identified at the C-terminus (amino-acids 2,038–2,167), corresponding to the Receptor Binding Protein (RBP) of *Salmonella* phage vB_senMS16 (Supplementary Figures 2, 3).

Our analysis returned very limited data for the five proteins encoded by the *orfs* between the *tal* and the *lysin* (tmp+8) genes, which usually may comprise the RBP, as well as baseplate ancillary proteins, which help maintaining the RBPs or providing additional binding domains. However, TMHMM (Käll et al., 2004) analysis reports that out of these five proteins, two contain one trans-membrane helix (TMP+5 and TMP+7) and one contains four trans-membrane helices (TMP+4). These three proteins are obvious candidates for a holin function.

**FIGURE 5:**
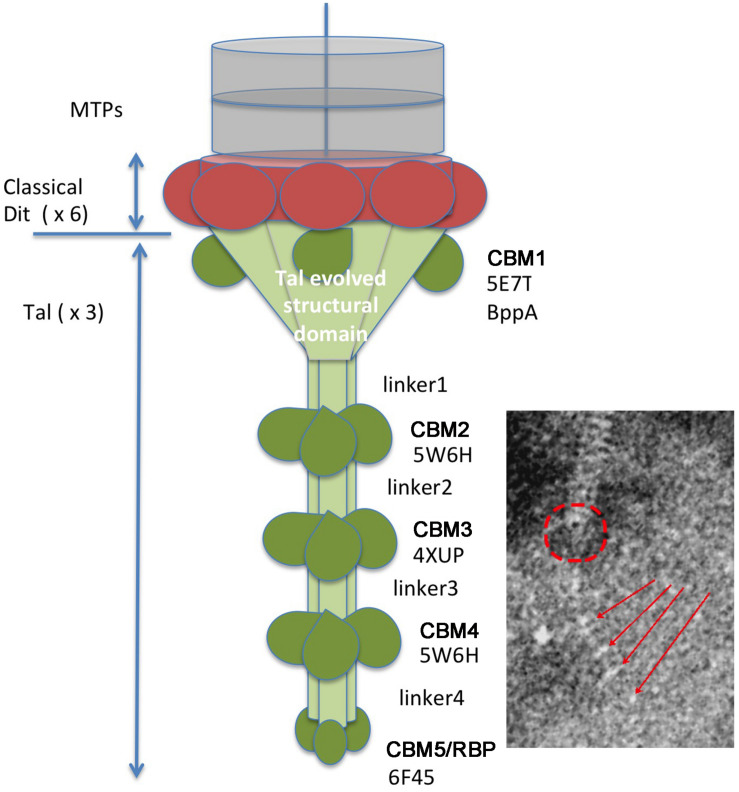
The proposed adhesion apparatus of Vinitor phages is based upon HHpred analysis. The classical Dit structural ring is colored in red. The Tal structural domains are colored in light green. The atypical CBM insertions (5 types) are colored in dark green. The putative location of the structural domain is shown by a red a circle and the 2–5 CBMs by red arrows on the EM micrographs. The Protein Data Bank identification numbers are displayed below each CBM label and identify the best HHpred hit.

Based on these HHpred reports, we established a topological model of Vinitor phages adhesion device (Figure 5). All 5 putative CBMs may participate in host adhesion. As we failed to identify any other CBM in the phage using HHpred, it is very likely that the tail extension analyzed here harbors the receptor binding modules and constitutes therefore the *bona fide* RBP. This is in contrast with the reports of the simultaneous presence of a Tal extension carrying CBMs and a RBP in *Moineauvirus* and *Brussowvirus S. thermophilus* phages (Lavelle et al., 2020). The fact that our current data are reflecting some diverging structure of the phage tail tip between Vinitor and other phages of LAB is not so surprising and is probably linked to the complex and specific outer structures of their bacterial hosts.

### Oenophage Phylogenies

The phylogenetic tree comparing the TerL proteins confirmed that Vinitor phages are only remotely related to other oenophages. They grouped with the dairy phages 5093 from *S. thermophilus* and LL-H from *L. delbrueckii* (Mikkonen and Alatossava, 1995), as well as (pro)phages collected from different sources such as phig1e from *L. plantarum* isolated from plant materials (Kodaira et al., 1997) and *C. intestini* (47% identity), a LAB isolated from the bumble bee gut (Praet et al., 2015). To better understand the evolutionary trajectory of the Vinitor phages, we built a second phylogenetic tree based on the MCP sequences (Figure 4B). This confirmed that the virulent oenophages are more related to the *pac*- than the *cos*-containing phages of *O. oeni*. Consistent with earlier observations was the clustering of Vinitor phages, LL-H from *L. delbrueckii*, a prophage from *C. intestini* and a short list of other LAB phages in a sister group. Phage LL-H and these latter phages shared the common feature to represent reference members of rare groups of phages/prophages infecting their cognate host species: Lj928 (*L. johnsonii*) (Ventura et al., 2004), 1358 (*Lac. lactis*) (Dupuis and Moineau, 2010) and P35 (*Listeria monocytogenes*) (Hodgson, 2000). These peculiar phages have been proposed to result from illegitimate recombination between dairy as well as non-dairy phages infecting distinct bacterial species. This has probably taken place in environments containing multiple bacterial genera and species, such as plants. Accordingly, phage P35 from *L. monocytogenes* has been isolated from silage (Hodgson, 2000). Along with this, the importance of living and decomposing plants as habitats for many LAB species is well demonstrated (Yu et al., 2020), and it is likely that epiphyte communities contribute to the emergence of novel LAB phages with shuffled gene organization. Genome mosaicism varies depending on the host, lifestyle, and genetic constitution of phages. Accordingly, Vinitor phages are probably evolving differently due to their specific environment eventhough the group shares common ancestors with other LAB phages. An interesting point worthy of further investigation is that grapes and grapevine are associated to many insects, including pest insects. The phylogenetic trees constructed using the whole nucleic acid sequences as well as the MCP and TerL proteins indicate that Vinitor phages and a prophage in *C. intestini* are more closely related to each other than to any other sequenced LAB phages (Figure 4). Of note, the closest homologs to gp7 (capsid scaffolding protein) and gp13 (major tail proteins) are also found associated with recently acquired and uncharacterized sequences from metagenomic investigations of insects. Taken together our analyses may provide somes clues to the possible origin of Vinitor phages, resulting from interactions between plant- and insect-related bacteria and their phages. These data also bring us closer to an explanation of the uniqueness of Vinitor phages, which may result from the current under-representation of phage proteins from insect-associated microbial communities in databases. Future exploration of this niche will probably assist phage classification of Vinitor phages, as the VICTOR analysis currently suggests that the group is distantly related to the LAB phages which have been recently classified into novel sub-families and genera in the ICTV database (Figure 4C).

## Conclusion

*Oenococcus* phages are understudied compared to other industrially relevant LAB phages. Our current study described a new phage species called Vinitor, which represents the first virulent phage in the *O. oeni* species. Vinitor phages differ markedly from other oenophages and LAB phages, in terms of their very low level of DNA sequence identity and the number of unique genes. Reconstruction of its evolutionary history is currently difficult. However, some structural similarities exist in the capsid and endolysin/holin proteins of other LAB phages, and also in the moron region, which suggest common ancestors for these LAB phages. In contrast, Vinitor phages have some specificities linked to their host, lifestyle and niche. They infect their bacterial host through highly specific tail tips determinants probably adapted to recognize specifically receptors at the surface of *O. oeni* that are still unknown. In addition, a few putative DNA replication elements show homologous ORFs in prophages infecting related species or genera (*Oenococcus*-*Weissella*), as well as a number of unidentified functions which seem phage-specific. This would suggest that some steps in the common mechanisms for the recruitment of the host replication machinery would be conserved among phages infecting members of the *Oenococcus* and *Weissella* genera. Furthermore, our study now urges for phage structural analysis and host cell wall biochemical studies in the genera *Oenococcus, Leuconostoc* and *Weisella* sp., to better understand phage-host interactions in these important group of food-associated LAB.

Further comprehensive study of how Vinitor phages can persist and possibly shape the indigenous population of *O. oeni* present on grapes will be of great value for understanding the early steps of spontaneous MLF fermentation. On the other hand, presence of virulent phages may bias the outcome of bacterial enrichment cultures, explaining the repeated difficulty in the isolation and cultivation of *O. oeni* strains from grapes (Lorentzen and Lucas, 2019).

## Data Availability Statement

The datasets presented in this study can be found in online repositories. The names of the repository/repositories and accession number(s) can be found in the article/Supplementary Material.

## Author Contributions

CP, AC, and FJ carried out the main body of research. FJ and CP collected the samples, isolated the phages, and performed the genome sequencing. CP, FJ, and OC performed bioinformatics analysis. PL contributed the bioinformatics analysis. AC performed isolates characterization with EM and host spectrum. JS contributed to the phage host spectrum experiments. CC ran the adhesion topology analysis. CP, AC, and CL wrote and edited the manuscript. CL supervised the work progress. All authors contributed to the article and approved the submitted version.

## Conflict of Interest

The authors declare that the research was conducted in the absence of any commercial or financial relationships that could be construed as a potential conflict of interest.

## Footnotes

1 https://pgtb.cgfb.u-bordeaux.fr

## Supplementary Material

Supplementary Figure 1 Alignment of Vinitor phages Tal proteins with MultAlin (Corpet, 1988).

Supplementary Figure 2 HHpred plots of Vinitor162 Tal proteins.

Supplementary Figure 3 Motifs in Vinitor27 Tal protein sequence.
