# Supplementary figures and images for: Characterization of the First Virulent Phage Infecting Oenococcus oeni, the Queen of the Cellars
Source: Front Microbiol. 2021 Jan 13;11:596541. doi: 10.3389/fmicb.2020.596541 (PMC7838156; doi:10.3389/fmicb.2020.596541)

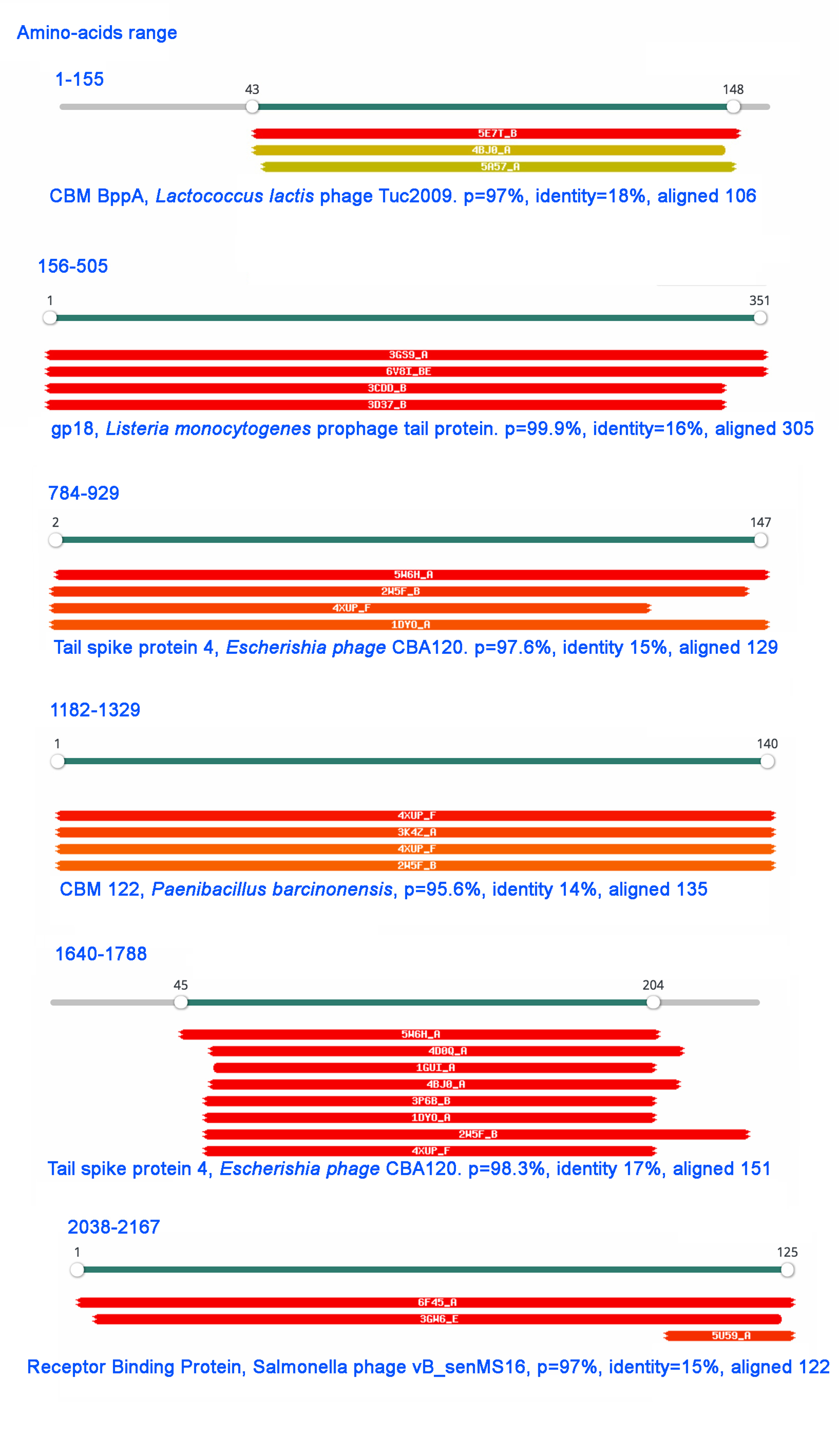

Supplement: Supplementary Figure 2 — HHpred plots of Vinitor162 Tal proteins. [file Image_2.TIF]

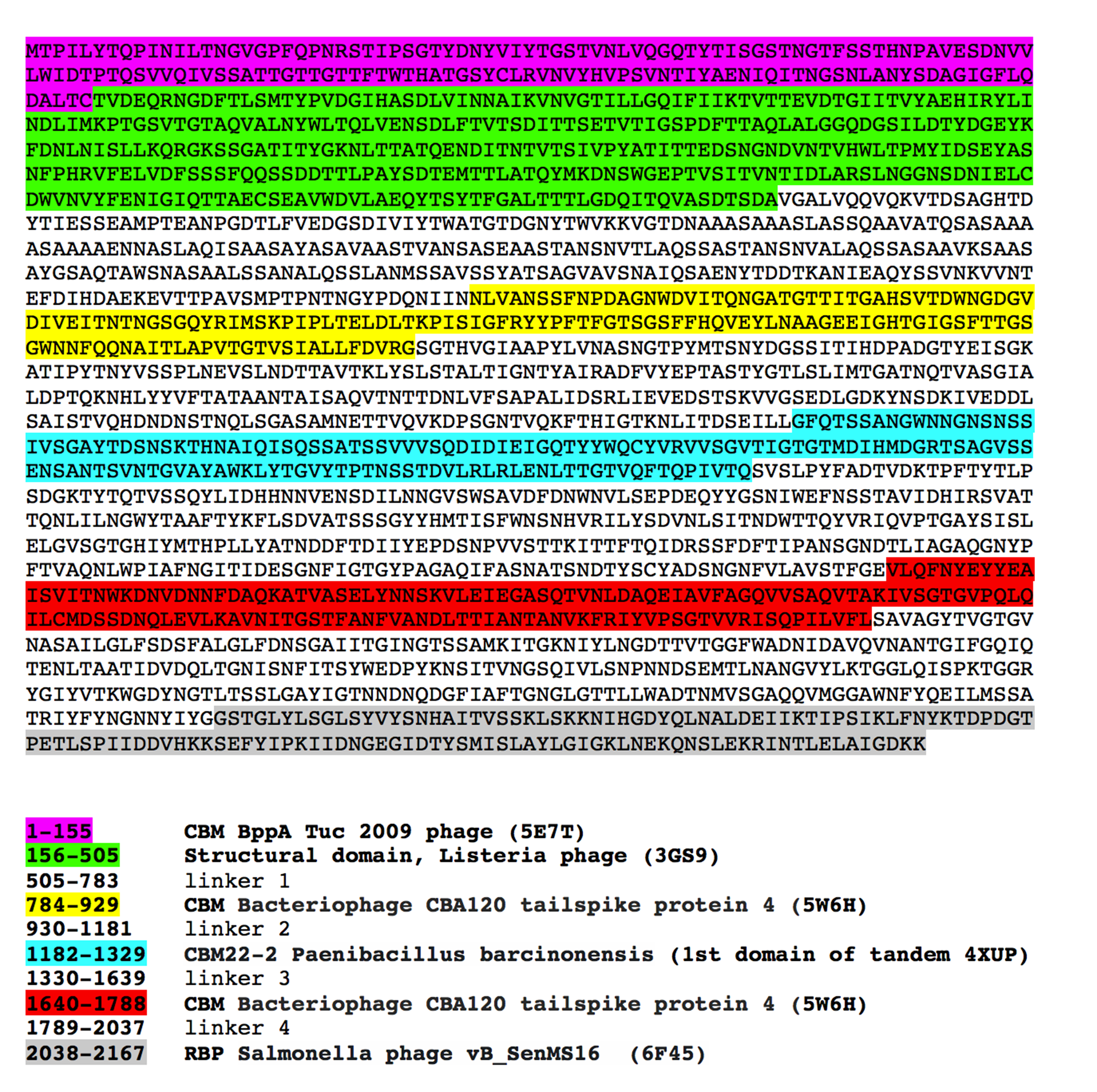

Supplement: Supplementary Figure 3 — Motifs in Vinitor27 Tal protein sequence. [file Image_3.TIFF]
