# Supplementary material for: Characterization of the First Virulent Phage Infecting Oenococcus oeni, the Queen of the Cellars
Source: Front Microbiol. 2021 Jan 13;11:596541. doi: 10.3389/fmicb.2020.596541 (PMC7838156; doi:10.3389/fmicb.2020.596541)
Supplement: Supplementary file 4 [file Data_Sheet_1.docx]

**Table S1.** Survey of oenophages during the 2015 vintage (50 positive samples/127)

| **W^a^** | **F^b^** | **Wine type** | **Grape**  **variety** | **Step** | **Nb^c^** | **oenophage group^d^** | | | | |
| --- | --- | --- | --- | --- | --- | --- | --- | --- | --- | --- |
|  |  |  |  |  |  | **Int_A_** | **Int_B_** | **Int_C_** | **Int_D_** | **0ther** |
| I | 1 | W | SauvG | post-ass | 128 | 128.2 |  |  |  |  |
|  | 2 | W | Sem | AF start | 12 | 12.2 |  |  |  | 12.2 |
|  |  |  |  | AF end | 26 |  | 26.1; 26.2 |  |  |  |
|  | 3 | W | Sauv | must | 5 |  |  |  |  | 5.1 |
|  |  |  |  | AF start | 10 |  | 10.2 |  |  |  |
|  | 4 | W | Musc | must | 9 |  |  |  |  | 9.1 |
|  |  |  |  | AF mid | 24 |  | 24.1 |  |  |  |
|  |  |  |  | AF end | 25 | 25.2 | 25.2 |  |  |  |
|  | 5 | R | Mer | must | 27 |  |  |  |  | 27.1 |
|  |  |  |  | MLF | 49 | 49.1 |  |  |  |  |
|  |  |  |  | MLF | 83 | 83.1 |  |  |  |  |
|  | 6 | R | Mer/CabF | post-ass | 85 | 85.1 |  |  |  |  |
|  | 7 | R | CabS | AF | 51 | 51.2 |  |  |  |  |
|  |  | R | CabS/Mer | post-ass | 84 | 84.1 |  |  |  |  |
|  | 8 | R | PetVer | MLF | 102 | 102.2 |  |  |  |  |
| II | 9 | R | Mer | AF end | 45 |  | 45.1 |  |  |  |
|  |  |  |  | Post-clar | 72 | 72.2 |  |  |  |  |
|  |  |  |  | MLF end | 76 |  | 76.1; 76.2 |  |  |  |
|  |  |  |  | Post-ass | 133 |  | 133.2 |  |  |  |
|  | 10 | R | CabS | MLF end | 77 |  | 77.2 |  |  |  |
|  | 11 | W | Sauv/Sem | AF | 15 | 15.2 |  |  |  |  |
|  | 12 | W | Sem | AF end | 29 | 29.2 |  |  |  |  |
| III | 13 | R | Mer  Conv | AF end | 47 | 47.1 |  |  |  | 47.1 |
|  |  |  |  | AF end | 53 | 53.2 |  |  |  |  |
|  |  |  |  | Post-clar | 75 |  | 75.1 |  |  |  |
|  |  |  |  | MLF | 96 |  | 96.2 |  |  |  |
|  |  |  |  | MLF | 97 |  | 97.2 |  |  |  |
|  |  |  |  | MLF | 107 |  | 107.2 | 107.2* |  |  |
|  |  |  |  | MLF | 108 |  | 108.1; 108.2 |  |  |  |
|  |  |  |  |  |  |  |  |  |  |  |
|  | 14 | R | Mer  Org | AF | 16 |  | 16.2 |  |  |  |
|  |  |  |  | AF end | 28 |  |  |  |  | 28.2 |
|  |  |  |  | MLF | 56 | 56.2 |  |  |  |  |
|  |  |  |  | MLF | 81 | 81.1 |  |  |  |  |
|  |  |  |  | MLF end | 82 |  | 82.2 |  |  |  |
|  |  |  |  | MLF end | 98 |  | 98.2 | 98.2* |  |  |
|  |  |  |  | MLF end | 99 | 99.2 |  |  |  |  |
|  |  |  |  | MLF end | 109 | 109.1 |  |  |  |  |
| IV | 15 | W | Sem | PDC | 17 |  | 17.2 |  |  |  |
| V | 17 | R | CabF | AF end | 78 |  | 78.1;78.2 |  |  |  |
| VI | 18 | R | Mer | AF end | 87 |  | 87.1; 87.2 |  |  |  |
|  |  |  |  | AF end | 88 |  | 88.1; 88.2 |  |  |  |
|  |  |  |  | AF end | 89 |  | 89.2 |  |  |  |
|  |  |  |  | MLF | 111 |  | 111.1;111.2 |  |  |  |
|  |  |  |  | MLF | 112 |  | 112.1 |  |  |  |
|  |  |  |  | MLF | 113 |  | 113.1 |  |  |  |
|  | 19 | R | CabF | AF end | 90 | 90.1; 90.2 |  |  |  |  |
|  |  |  |  | MLF | 95 | 95.1 |  |  |  |  |
|  |  |  |  | MLF end | 104 | 104.1 |  |  |  |  |
|  | 20 | R | CabF | MLF | 114 |  | 114.1; 114.2 |  |  |  |
|  | 21 | R | CabS | AF end | 91 |  | 91.1 |  |  |  |
| Total phage lysates (64) | | | | | | 22 | 34 | 2* | 0 | 6 |

^a^ Winery ; ^b^ Fermentation tank ; ^c^ sample number ; ^d^ typing was done by PCR for the temperate oenophages as previously described (Jaomanjaka et al., 2013). Phages showing absence of signals were classified as « others ».

W, white wine ; R, red wine ; SauvG, Sauvignon Gris ; Sem, Semillon ; Sauv, Sauvignon ; Musc, Muscadelle ; Mer, Merlot ; CabF, Cabernet Franc ; CabS, Cabernet Sauvignon ; Conv, conventional production ; Org, organic production ; post-ass, post-assembly ; AF, alcoholic fermentation ; MLF, malo-lactic fermentation ; post-clar, post-clarification. The asterisk indicated that Int_C_ phages could not be isolated as pure lysates.

**Table S2.** Host range analysis of phage Vinitor162 against different strains of LAB species

| **Species** | **Name** | **Origin**  **/commercial name** | **EOP** |
| --- | --- | --- | --- |
| *Lactobacillus crispatus* | 112.59A; 107.7B; 100.16A | Vaginal isolates (MIVEGEC, U. Montpellier, CNRS, IRD) | <10^-8^ |
| *Lactobacillus gasseri* | 100.7A; 114.1A; 117.75A |  | <10^-8^ |
| *Lactobacillus johnsonii* | 37.31B; 113.22A |  |  |
| *Lacticaseibacillus rhamnosus* | A23; 51A; 57A1; 46A; 52B; 24A; 35A; A22; A21; RZD2; 27C; 21B; 25A | Buccal isolates (CRBOeno) | <10^-8^ |
| *Lactiplantibacillus plantarum* | BMS2 | Buccal isolates (CRBOeno) | <10^-8^ |
| *Limosilactobacillus fermentum* | BAP3 | Buccal isolates (CRBOeno) | <10^-8^ |

EOP, efficiency of plating. Strain IOEB277 has an EOP of 1.

**Table S3.** Vinitor162 genome annotation

| Gene | Start-end | Putative function | Length (AA) | Percent identity* | e-value | Source (BG, bacterial genome or P, phage) | GenBank accession number |
| --- | --- | --- | --- | --- | --- | --- | --- |
| 1 | 30-584 | Terminase, small subunit | 184 | 33 (55-177) | 4e-10 | BG, *Lactobacillus* sp.YH-lac9 | WP_172188902 |
| 2 | 565-1869 | Terminase, large subunit | 434 | 51 (3/433) | 1e-152 | BG, *Limosilactobacillus mucosae* | KGL66837 |
| 3 | 1869-3485 | Putative phage portal protein | 538 | 41 (1/499) | 3e-122 | P*, Latilactobacillus sakei* subsp. *sakei* | RXA80305 |
| 4 | 3485-3688 | HP | 67 | - | - | - | - |
| 5 | 3757-4755 | Minor capsid protein | 332 | 36 (9/311) | 2e-56 | BG*, Weissella* sp.8H-2 | WP_148623045.1 |
| 6 | 4755-5039 | HP | 94 | - | - | *-* | - |
| 7 | 5254-5859 | Phage scaffolding protein | 201 | 44 (1-167)  44 (1-170) | 7e-33  1e-29 | BG, *Lacticaseibacillus songhuajiangensis*  P, insect metagenome | WP_125570919.1  QHJ82409 |
| 8 | 5910-7016 | Major capsid protein | 368 | 57 (168/297) | 2e-118 | BG, *Convivina intestini* | WP_089940226.1 |
| 9 | 7097-7823 | Putative connector | 132 | 36 (48/134) | 2e-19 | BG, *Lb.spp* | WP_125650834.1 |
| 10 | 7479-7823 | Minor capsid protein | 114 | 37 (4/114)  37 (41/112) | 1e-15  4e-13 | BG, *Streptococcus pneumoniae*  P, phage protein Gp9, *S.suis* | WP_160545173.1  WP_044981543 |
| 11 | 7823-8236 | Capsid protein | 137 | 39 (51/131) | 8e-23 | BG, *L.monocytogenes* | MCQ88028.1 |
| 12 | 8223-624 | Minor capsid protein | 133 | 35 (35/100)  31 (6/132) | 2e-08  4e-08 | BG, Atopobacter sp.  P, *Lc. lactis* | WP_121565380  KSU09196.1 |
| 13 | 8635-9408 | Major tail protein with Ig-like domain | 257 | 40 (97/242)  37 (16/141) | 7e-35  2e-14 | BG, *Paucilactobacillus vaccinostercus*  P, insect metagenome | KRM61031.1  QHJ82476.1 |
| 14 | 9424-9780 | HP | 118 | 37 (42/113) | 6e-13 | BG, *Weisella confusa* | WP_161691374.1 |
| 15 | 9773-19390 | HP | 205 | 31 (63/203) | 8e-25 | P, Gp15 phage*, Lc. lactis* | WP_058225089.1 |
| 16 | 10380-14204 | Tape Measure Protein | 1274 | 53 (74/1137) | 0 | BG, *O.oeni* | WP_050496444 |
| 17 | 14201-14932 | Distal Tail protein (Dit) | 243 | 35 (1-242) | 2e-37 | BG, *W. confusa* | WP_161691380.1 |
| 18 | 14929-21441 | Tail associated Lysin (Tal) | 2170 | 36 (137-801; 1778-1952) | 1e-87 | BG, *W. confusa* | WP_161691382.1 |
| 19 | 21438-21623 | HP | 61 | - | - | - | - |
| 20 | 21604-21996 | HP | 130 | - | - | - | - |
| 21 | 21993-22433 | HP | 146 | - | - | - | - |
| 22 | 22476-22802 | HP | 108 | - | - | - | - |
| 23 | 22879-23412 | Putative holin | 177 | 27 (1/109) | 3e-06 | P, LL-H; *Lactobacillus* | AAC00556 |
| 24 | 23412-24503 | Lysin (muramidase) | 362 | 53 (1-360) | 1e-118 | BG, *O. sicerae* | WP_128686739.1 |
| 25 | 24867-24541 | VRR-NUC domain-containing protein | 108 | 46 (50/108) | 9e-23 | BG, *Lactiplantibacillus plantarum* | WP_069302375.1 |
| 26 | 25101-25394 | HP | 97 | - | - |  |  |
| 27 | 25391-26233 | Putative replication protein | 280 | 32 (95/299)  29 (87/296) | 9e-365  e-28 | BG, *O. sicerae*  P, *Enterococcus* phage phiEf11 | WP_128686704.1YP_003358835.1 |
| 28 | 26226-26915 | DUF1071 domain-containing HP | 229 | 45 (104/231)   \|  \|  \| \| --- \| --- \| | 7e-59 | BG, *Ent. faecium* | WP_086324935.1 |
| 29 | 26915-27097 | HP | 60 | - | - | *-* | - |
| 30 | 27094-27990 | Phage replisome organizer | 298 | 50 (1/254) | 3e-70 | BG, *W. fabalis* | WP_168721271.1 |
| 31 | 27980-28612 | HP, conserved among oenophages | 210 | 62 (1/210) | 4e-85 | P, 0E33PA, *O.oeni* | AWT48049.1 |
| 32 | 28609-28776 | HP | 55 | 95 (52/55) | 9e-26 | BG, *O. oeni* | WP_096860438.1 |
| 33 | 28773-29201 | YopX-like protein (PfamPF09643) | 142 | 74 (102/137)  36 (3/137) | 2e-59  1e-12 | BG, *O. oeni*  P, uncult. Mediterranean phage | WP_071428701.1  ANS04906.1 |
| 34 | 29202-29351 | HP | 49 | - | - | *-* | - |
| 35 | 29332-29691 | HP | 119 | 38 (42/112)   \|  \|  \| \| --- \| --- \| | 6e-16 | BG, *O. ciserae* | WP_128686703.1L |
| 36 | 29688-30041 | HP | 117 | 31 (26/85) | 2e-4 | BG, *O. oeni* | WP_071439831.1 |
| 37 | 30063-30257 | HP | 64 | - | - | *-* | - |
| 38 | 30257-30613 | HP | 118 | 42 (30/71)  40 (23/87) | 5e-05  8e-04 | BG, *Leptotrichia massiliensis*  P, viral metagenome ocean, Tara | WP_083378248.1  QDP50148.1 |
| 39 | 30838-31158 | HP | 106 | - | - | *-* | - |
| 40 | 31158-31397 | HP | 79 | - | - | *-* | - |
| 41 | 31399-31605 | HP | 68 | - | - | *-* | - |
| 42 | 31595-31816 | HP | 73 | 56 (1/73)  56 (41/73) | 3e-19  3e-18 | BG, *O. oeni* isolates (Australia)  BG, mine drainage isolate | RWZ78830.1  AVI93419.1 |
| 43 | 31816-32055 | HP | 79 | - | - | *-* | - |
| 44 | 32052-32255 | HP | 67 | - | - | *-* | - |
| 45 | 32252-32566 | HP | 104 | - | - | *-* | - |
| 46 | 32559-33005 | HP | 148 | - | - | *-* | - |
| 47 | 33002-33442 | HP | 146 | - | - | *-* | - |
| 48 | 34165-34776 | Chrom. partitioning prot. Spo0J | 203 | 47 (29-200)  45 (34-203) | 3e-38  2e-34 | BG, *Lactobacillus helveticus*  P, *Lactococcus* phage P335, orf23 | WP_046814054.1 |
| 49 | 34755-35432 | Putative ABC-transporter ATPase | 225 | 67 (1-225)  62 (140/226) | 8e-110  1e-89 | BG, *Ligilactobacillus salivarius*  P, *Lactococcus* phage P335, orf24 | WP_081510396.1  ABI54227.1 |
| 50 | 35429-35815 | Putative N-acetyltransferase | 128 | 50 (64/127)  44 (56/126) | 6e-36  3e-29 | BG, *S. pyogenes*  P, *Lactococcus* phage P335, orf25 | WP_080262024.1  ABI54228.1 |
| 51 | 35905-36285 | ASCH domain-containing HP | 130 | 44 (55/124) | 2e-30 | BG, *Bacillus* sp. | WP_014480932.1 |

HP: hypothetical protein
